# Prevalence of *pfdhfr* and *pfdhps* mutations in *Plasmodium falciparum* associated with drug resistance among pregnant women receiving IPTp-SP at Msambweni County Referral Hospital, Kwale County, Kenya

**DOI:** 10.1186/s12936-020-03263-z

**Published:** 2020-05-24

**Authors:** Stella Wanjiku Gikunju, Eric L. Agola, Raphael Omusebe Ondondo, Johnson Kinyua, Francis Kimani, Angelle Desiree LaBeaud, Indu Malhotra, Charles King, Kelvin Thiong’o, Francis Mutuku

**Affiliations:** 1 Jomo Kenyatta University of Agriculture and Technology (JKUAT), Nairobi, Kenya; 2 Kenya Medical Research Institute (KEMRI), Centre for Biotechnology Research and Development (CBRD), Nairobi, Kenya; 3 Masinde Muliro University of Science and Technology, Kakamega, Kenya; 4 Department of Pediatrics, Division of Infectious Disease, Stanford University School of Medicine, Stanford, CA USA; 5 Center for Global Health and Diseases, Case Western Reserve University, Cleveland, OH USA; 6 Department of Environment and Health Sciences, Technical University of Mombasa, Mombasa, Kenya; 7 Technical University of Kenya, Nairobi, Kenya

**Keywords:** *Plasmodium falciparum*, Drug resistance, SP, *Pfdhfr*, *Pfdhps*, IPTp-SP, Pregnant women, Malaria

## Abstract

**Background:**

Prevention and treatment of malaria during pregnancy is crucial in dealing with maternal mortality and adverse fetal outcomes. The World Health Organization recommendation to treat all pregnant women with sulfadoxine-pyrimethamine (SP) through antenatal care structures was implemented in Kenya in the year 1998, but concerns about its effectiveness in preventing malaria in pregnancy has arisen due to the spread of SP resistant parasites. This study aimed to determine the prevalence of SP resistance markers in *Plasmodium falciparum* parasites isolated from pregnant women seeking antenatal care at Msambweni County Referral Hospital, located in coastal Kenya, between the year 2013 and 2015.

**Methods:**

This hospital-based study included 106 malaria positive whole blood samples for analysis of SP resistance markers within the *Pfdhfr* gene (codons 51, 59 and 108) and *Pfdhps* gene (codons 437 and 540). The venous blood collected from all pregnant women was tested for malaria via light microscopy, then the malaria positive samples were separated into plasma and red cells and stored in a − 86° freezer for further studies. Archived red blood cells were processed for molecular characterization of SP resistance markers within the *Pfdhfr* and *Pfdhps* genes using real time PCR platform and Sanger sequencing.

**Results:**

All samples had at least one mutation in the genes associated with drug resistance; polymorphism prevalence of *Pfdhfr*51**I**, 59**R** and 108**N** was at 88.7%, 78.3% and 93.4%, respectively, while *Pfdhps* polymorphism accounted for 94.3% and 91.5% at 437**G** and 540**E**, respectively. Quintuple mutations (at all the five codons) conferring total SP resistance had the highest prevalence of 85.8%. Quadruple mutations were observed at a frequency of 10.4%, and 24.5% had a mixed outcome of both wildtype and mutant genotypes in the genes of interest.

**Conclusion:**

The data suggest a high prevalence of *P. falciparum* genetic variations conferring resistance to SP among pregnant women, which may explain reduced efficacy of IPTp treatment in Kenya. There is need for extensive SP resistance profiling in Kenya to inform IPTp drug choices for successful malaria prevention during pregnancy.

## Background

Malaria is a significant public health problem in sub-Saharan Africa and remains a major contributor to morbidity and mortality in the African continent [1]. The World Health Organization (WHO) has reported that Africa carries the highest burden of malaria with 92% of the malaria cases and 93% of malaria deaths worldwide [2]. 99.7% of the cases are caused by *Plasmodium falciparum* with pregnant women being a particularly vulnerable population, especially those carrying first pregnancies at a young maternal age. Malaria in pregnancy contributes to maternal anaemia leading to spontaneous abortion, stillbirth, premature birth and low birth weight [2–4]. The WHO has recommended an intermittent preventative treatment for pregnant women (IPTp) interventions using sulfadoxine-pyrimethamine (SP) that Kenya implemented in the year 1998. Pregnant women received at least two doses of SP given from the second trimester of pregnancy, which was later revised in 2009 to a monthly dose administered during their antenatal clinic (ANC) visits [5].

IPTp prophylactic treatment has quickly been countered by the rise of *P. falciparum* parasites resistant to SP, resulting in the loss in sensitivity to the SP drug. This resistance is attributed to single nucleotide polymorphism (SNP) mutations within the *dhfr* and *dhps* genes that are target sites for the pyrimethamine and sulfadoxine active components of the drug, which are most effective when working in synergy [6]. In East Africa, the prevalence of these mutations is high, reaching near 100% in some regions [7–10], thus raising concerns on the efficacy of the drug in preventing malaria in pregnancy.

SP is still considered by practitioners to be effective in clearing the parasites in pregnant women, despite the high levels of *P. falciparum* resistance that have been reported. To clarify on this issue, the WHO recommended that more studies be carried out to investigate the prevalence of *P. falciparum* SP resistance molecular markers in the context of IPTp [11], which formed the main objective of this study in Kwale county. Malaria is the leading cause of morbidity in this county with a prevalence of 37.7% in comparison to other disease morbidities, such as influenza, diarrhoea, and respiratory diseases among others, which account for 16.4, 4.6 and 5 per cent of disease burden in the county, respectively [12].

This study investigated the prevalence of SP resistance molecular markers in parasites isolated from blood samples collected from the pregnant women receiving IPTp-SP treatment between 2013 and 2015 at Msambweni County Referral Hospital in Kwale County, Kenya. Since SP is the recommended drug for IPTp, continuous monitoring of its efficacy and for *P. falciparum* resistance molecular markers is key in addressing malaria control among pregnant women. This study is among the first to assess resistance markers among pregnant women in Kwale coastal Kenya, contributing invaluable data on the rising prevalence of SP resistant *P. falciparum* among pregnant women in Kenya.

## Methods

### Study population and sample collection

Kenya endosed the WHO policy to perform a malaria test for every pregnant woman visiting the antenatal clinic from the first visit to time of delivery in the year 2009 [12]. In this hospital based cross-sectional study, a total of 763 pregnant women sort antenatal care at the Msambweni County Referral Hospital between the year 2013 and 2015 and intravenous blood was collected and screened for malaria parasites via light microscopy. The first malaria blood sample was taken immediately after enrolment of the mother and before administration of the first SP-IPTp dose. SP was then administered at each monthly visit and the pregnant women were treated for any symptomatic intercurrent malaria infection with artemether-lumefantrine [13]. Parasite density was determined for malaria positive samples using a previously published protocol [14] and the whole blood components separated into plasma and red cells. The latter was transferred into 2 ml cryovials and archived at − 86 °C.

Demographic data was drawn from all participants to assess the risk factors to malaria infection. In this study, data such as age, gravidity, SP dosage issued at every IPTp visit and other infections leading to anaemia were analysed.

Whatman filter paper was used to prepare dried blood spots (DBS) from *P. falciparum* positive archived red blood cell samples, which were then individually preserved in coded plastic bags with silica desiccant beads. The DBS’ were transported at room temperature to Centre for Biotechnology Research and Development (CBRD) within the Kenya Medical Research Institute (KEMRI) in Nairobi, Kenya for further molecular analysis.

### DNA extraction and RT-PCR genotyping

Genomic DNA was extracted from the DBS using a QIAamp DNA mini blood kit (Qiagen, Hilden, Germany), following the manufacturer’s instructions. DNA was eluted with AVE buffer at a volume of 100 µl and stored at − 80 °C. A singleplex real-time PCR assay carried out on an Applied Biosystems™ 7500 Fast Real-time PCR machine was used to confirm all samples for *P. falciparum* [15] with each sample denatured at 95 °C for 10 s and cycled 45 times with each cycle consisting of 95 °C for 15 s and 55 °C for 60 s [16].

The presence of mutations in *Pfdhfr* (codons 51, 59,108) and *Pfdhps* (codons 437, 540) genes associated with SP drug resistance were assessed using a modified multiplex real time PCR assay previously described [16]. The RT-PCR mastermix reaction of 25 µl final volume contained 12.5 µl of Agpath-ID™ One-Step RT-PCR Kit, 5 µl of DNA template, forward and reverse primers at various concentrations and two hydrolysis probes for each codon at a final concentration of 0.2 µM; A FAM labelled probe to detect the wildtype strain and a HEX labelled probe to detect the mutant strain. Both probes were tagged to a Black hole quencher (Table 1). All PCR primers and probes were synthesized by Macrogen Inc. (Seoul, Korea) and thermocyclic conditions were set as those for the singleplex assay.

**Table 1:** Details of Primers used in genetic evaluation of Pfdhfr and Pfdhps SNPs in Plasmodium falciparum isolates from Msambweni County Referral Hospital, Kwale, Kenya

No.	Primer name	5′ end	Sequence	3′ end	Amount	Modified Oligo
1	P.falc F	5´	GCTCTTTCTTGATTTCTTGGATG	3´	0.3 uM	No
2	P.falc R	5´	AGCAGGTTAAGATCTCGTTCG	3´	0.3 uM	No
3	P.falc P	5´	CACGAACTAAAAACGGCCAT	3´	0.2 uM	FAM-BHQ1
4	DHFR-51 F	5´	TGAGGTTTTTAATAACTACACATTTAGAGGTCT	3´	0.3 uM	No
5	DHFR-51 R	5´	TATCATTTACATTATCCACAGTTTCTTTGTT	3´	0.3 uM	No
6	DHFR-51 WTP	5´	AATGTAATTCCCTAGATATG	3´	0.2 uM	FAM-BHQ1
7	DHFR-51 MP	5´	AAATGTATTTCCCTAGATATG	3´	0.2 uM	HEX-BHQ1
8	DHFR-59 F	5´	TGAGGTTTTTAATAACTACACATTTAGAGGTCT	3´	0.3 uM	No
9	DHFR-59 R	5´	TATCATTTACATTATCCACAGTTTCTTTGTT	3´	0.3 uM	No
10	DHFR-59 WTP	5´	AATATTTTTGTGCAGTTACA	3´	0.2 uM	FAM-BHQ1
11	DHFR-59 MP	5´	TGAAATATTTTCGTGCAGTTA	3´	0.2 uM	HEX-BHQ1
12	DHFR-108 F	5´	TGGATAATGTAAATGATATGCCTAATTCTAA	3´	0.3 uM	No
13	DHFR-108 R	5´	AATCTTCTTTTTTTAAGGTTCTAGACAATATAACA	3´	0.3 uM	No
14	DHFR-108 WTP	5´	AGAACAAGCTGGGAAA	3´	0.2 uM	FAM-BHQ1
15	DHFR-108 MP	5´	AGAACAAACTGGGAAAG	3´	0.2 uM	HEX-BHQ1
16	DHPS-437 F	5´	TGAAATGATAAATGAAGGTGCTAGTGT	3´	0.9 uM	No
17	DHPS-437 R	5´	AATACAGGTACTACTAAATCTCTTTCACTAATTTTT	3´	0.9 uM	No
18	DHPS-437 WTP	5´	AGAATCCTCTGCTCCT	3´	0.2 uM	FAM-BHQ1
19	DHPS-437 MP	5´	AATCCTCTGGTCCTTT	3´	0.2 uM	HEX-BHQ1
20	DHPS-540 F	5´	AATGCATAAAAGAGGAAATCCACAT	3´	0.3 uM	No
21	DHPS-540 R	5´	TCGCAAATCCTAATCCAATATCAA	3´	0.3 uM	No
22	DHPS-540 WTP	5´	CAATGGATAAACTAACAAA	3´	0.2 uM	FAM-BHQ1
23	DHPS-540 MP	5´	AATGGATGAACTAACAAA	3´	0.2 uM	HEX-BHQ1

*Plasmodium falciparum* laboratory strains 3D7, Dd2, V1/S and W2 were used as positive controls for wildtype and mutant strains. Samples were considered positive for either wildtype or mutant strain if they amplified with a PCR cycle threshold (CT) value within 40 cycles. Mixed outcomes were identified as those with both wildtype and mutant PCR amplifications.

### Conventional PCR and Sanger sequencing

For quality control, samples with mixed outcome were amplified and further differentiated using nested PCR as previously described [17]. The PCR products were separated with electrophoresis on a 1.5% agarose gel, stained with ethidium bromide and visualized under UV transillumination using a UVP GelDoc-It^®^2 Imager. The amplified products were sequenced by the Inqaba Biotec company using the ABI 3500XL Genetic analyser. Mutations on nucleotide sequences were examined using BioEdit 7.0 and the reference sequences of the *Pfdhfr* gene (PF3D7_0417200) and the *Pfdhps* gene (PF3D7_0810800) were acquired from NCBI.

### Statistical analysis

Characteristics of women were presented as means with standard deviations (SD) and as proportions. The prevalence of malaria parasites and mutations were expressed as a proportion with their respective 95% confidence interval (95% CI), while parasite load as median and its interquartile range (IQR). Comparisons of different factors for significant difference was done using *t* test for quantitative variables while Chi square was used for binary variables, with a p-value of < 0.05 considered significant. Statistical analysis was conducted using Stata version 12.0 software (StataCorp, 4905 Lakeway Drive, College Station, Texas 77845 USA).

## Results

The 763 pregnant women screened for malaria in this study had a mean (± SD) age and gestation of 26 (± 6.4) years and 23 (± 5.2) weeks, respectively. The median (IQR) number of visits with dispensed folic tablets and SP-IPTp were three (3–4) and three (2–4), respectively. The majority of women (98%) had at least one dose of IPTp, while 88% received the WHO recommended IPTp doses (≥ 2). Malaria parasites were detected in 135 pregnant women, yielding a prevalence of 17.7% (95% CI 15.1–20.6). Young age and first pregnancy were significantly associated with malaria parasite infection (Table 2). Primigravidae were 1.7-times as likely to be detected with malaria parasites compared to multigravidae (OR = 1.7; 95%CI 1.1–2.5).

**Table 2:** Characteristics of pregnant women screened for malaria parasites between 2013–2015 at Msambweni County Referral Hospital in Kwale, Kenya

Characteristic	Negative (N = 628)	Positive (N = 135)	P value
Age (mean; SD) years	26.4 (6.31)	24.4 (6.34)	<0.001
Education (n, %)			
None to lower primary	149 (24%)	41 (30%)	Ref
Upper primary	358 (57%)	72 (53%)	0.185
Secondary	121 (19%)	22 (16%)	0.198
Occupation (n, %)			
Unemployed	541 (86%)	123 (81%)	Ref
Employed (formal or self)	87 (14%)	12 (9%)	0.157
Monthly household expenditure (n, %)			
< 5000	45 (7%)	12 (9%)	Ref
≥ 5000	583 (93%)	123 (91%)	0.610
Bed net (n, %)			
Yes	596 (95%)	122 (90%)	Ref
No	32 (5%)	13 (10%)	0.067
First pregnancy (n, %)			
Yes	144 (23%)	45 (33%)	Ref
No	484 (77%)	90 (67%)	0.015
Gestation in weeks (mean, SD)	23.3 (5.17)	22.1 (5.21)	0.017
Number of Visits of Folic use (mean, SD)	3.4 (1.39)	3.8 (1.36)	0.007
Number of Visits of Fancida use (mean, SD)	3.2 (1.39)	3.5 (1.39)	0.015
HB g/dl (mean, SD)	9.8 (1.82)	9.9 (1.57)	0.472
Parasite load (median; IQR)	NA	2400 (960-7200)	NA

Of the 568 women with data on haemoglobin levels, the mean Hb was 9.8 (± 1.78) g/dL and 425 (75%) were anaemic (Hb ≤ 11.0 g/dL). In 277 women with anaemia and that had other investigations, 30 (11%) had hookworm infection while only three of 99 women without anaemia were infected. Therefore, hookworm infection was significantly associated with a near fourfold increased odds of anaemia among these pregnant women (OR = 3.9; 95%CI 1.2–13.0), but not infection with malaria parasites (p = 0.699). Women with hookworm infection had a significantly lower mean Hb (9.3 ± 1.38 g/dL) compared to those negative for hookworm (10.0 ± 1.78 g/dL, p < 0.01).

Of the 135 pregnant women that tested positive for malaria, only 84 had archived blood samples available and these were included in this study’s genetic analysis. Table 3 present socidemographic characteristics of these 84 women. A total of 106 blood samples (70 blood samples collected at first antenatal care visit and 36 collected at delivery) were confirmed to contain *P. falciparum* parasites using a singleplex real time PCR assay and were included in the mutation analyses. Unfortunately, the number of women that consistently sort antenatal care from first visit to delivery at the Msambweni hospital were few therefore this study could not draw conclusive analysis on recrudescence malaria infections. Each sample collected was considered unique for analysis of prevalence of SP resistant markers. The median parasite load was 2760 (1200–7133) parasites/µL of blood.

**Table 3:** Characteristics of pregnant women from Msambweni Country referral hospital, Kwale Kenya from whom *Plasmodium falciparum* isolates were recovered for analysis (N = 84)

Characteristic	Mean, median or proportion
Age in years (mean; SD)	23.7 (6.14)
Marital status	
Single	19 (23%)
Married	53 (63%)
Cohabiting	11 (13%)
Divorced	1 (1%)
Education	
None to lower primary school	24 (29%)
Upper primary school	48 (57%)
Secondary school	12 (14%)
Occupation	
Unemployed	79 (94%)
Employed	5 (6%)
Monthly household expenditure (KShs.):	
< 5000	61 (73%)
≥ 5000	23 (27%)
Bed net use	
Yes	76 (90%)
No	8 (10%)
First pregnancy?	
Yes	32 (38%)
No	52 (62%)
Gestation in weeks (mean, SD)	22.0 (5.19)
Number of Visits of Folic acid use (mean, SD)	3.7 (1.40)
Number of Visits of Fansidar use (mean, SD)	3.5 (1.43)
Hb g/dL (mean, SD)	9.5 (2.31)
Parasite load/µL of blood (median; IQR)	2760 (1200–7133)

All samples were successfully genotyped yielding prevalences of *Pfdhfr* gene and *Pfdhps* SNP mutations that ranged from 83–100% (Table 4a). Of the 106 genotypes, 94 (88.7%), 83 (78.3%) and 99 (93.4%) harboured *Pfdhfr* gene mutant allele 51**I,** 59**R** and 108**N** with a total of 33 samples having mixed outcomes at these alleles (Table 4a) The overall frequency of parasites with the *Pfdhfr* triple mutant genotype was 87.4%. In estimating the prevalence of mutations observed in the *Pfdhps* gene, 94.3% and 91.5% of the 106 samples harboured the mutant allele 437**G** and 540**E**, respectively, with 5 samples having a mixed outcome The overall frequency of mutations in the *Pfdhps* gene was at 94.4%.

**Table 4:** Genetic analysis of *Pfdhfr* and *Pfdhps* SNPs in *Plasmodium falciparum* isolates collected from pregnant women at Msambweni County Referral Hospital in Kwale County, Kenya

SNP (N = 106)	Mutant type n (%, 95% CI)	Mixed type n (%, 95% CI)	Wild type n (%, 95% CI)
a Prevalence of *Pfdhfr* and *Pfdhps* SNPs in isolated *P. falciparum malaria parasites*			
51	94 (88.68, 80.69 to 93.76)	6 (5.66, 2.32 to 12.41)	5 (4.72, 1.75 to 11.19)
59	83 (78.30, 69.03 to 85.48)	21 (19.81, 12.95 to 28.91)	2 (1.89, 0.33 to 7.32)
108	99 (93.40, 86.40 to 97.08)	6 (5.66, 2.32 to 12.41)	0 (0.00)
437	100 (94.34, 87.59 to 97.68)	4 (3.77, 1.21 to 9.94)	2 (1.89, 0.33 to 7.32)
540	97 (91.51, 84.07 to 95.80)	1 (0.64, 0.05 to 5.90)	5 (4.72, 1.75 to 11.19)

Genotype	Mutants only		Mutants + Mixed	
	N	%	N	%
b Different combinations of *PfDHFR/PfDHPS* haplotype mutations				
Dup	7	6.6	0	0
Tri	7	6.6	4	3.8
Quad	22	20.8	11	10.4
Quint	70	66	91	85.8

In combination of *Pfdhfr and Pfdhps* haplotypes (Table 4b), quintuple mutant genotype (51**I** + 59**R** + 108**N**) + (437**G** + 540**E**) was the most prevalent at 85.8% (91/106). Quadruple mutations [(51**I** + 59**R **+ 108**N**) + 437**G**] or [(59**R** + 108**N**) + (437**G** + 540**E**)] were observed at 10.4% (11/106), where five of the samples had the wildtype allele at codon **N**51. Triple mutation (51**I** + 59**R** + 108**N**)  was the least prevalent at 3.8% (4/106) where mutant polymorphisms were in the *Pfdhfr* gene and wildtype alleles were present in the *Pfdhps* gene. While some wildtype alleles were observed at *Pfdhps* gene, no sample was fully wildtype at the *Pfdhfr* gene. The samples with mixed outcomes were confirmed for mutant alleles using Sanger sequencing and considered as mutant. Clinically, 72 (86%) of the 84 pregnant women had quintuple genotype (i.e. had pure or mixed mutations at all the five loci) suggesting total resistance to SP among these women. Only eight and four women had quadruple and triple genotypes, respectively.

## Discussion

Malaria prevalence of 18% found in this study was higher than 10% and 13% observed among pregnant women in another Kenyan coastal region [18] and the Lake region in Tanzania ([19], respectively, but significantly lower than 31% documented in the Kenyan Lake region [18]. The high burden of malaria in pregnancy remains of public health concern as observed in this study where younger women in their first pregnancy were at greatest risk of infection. Kwale county is known to be both a malaria and lymphatic filariasis endemic zone [20] and a four fold increase of anaemia among the pregnant women resulting from hookworm infections was a significant finding impacting on the health of pregnant women.

IPTp-SP is an important prophylactic therapy recommended for prevention of malaria in high endemic African regions and the emerging high *P.falciparum* resistance to the SP is likely to render it ineffective. In this study, a prevalence of 85.8% quintuple mutations that confer to SP resistance was reported. This is a great increase from 53.7% quintuple mutations identified among children seeking medical care at the same hospital where this study was conducted [21]. Similarly, studies conducted in western Kenya and Uganda reported high prevalence (78–97%) of quintuple *Pfdhfr/Pfdhps* haplotype mutations [8, 10], with 85–100% polymorphisms observed within the *Pfdhfr* 51**I**, 59**R** and 108**N** which compares to 89, 78 and ≥ 97% polymorphisms observed at *Pfdhfr* 51**I**, 59**R** and 108**N** in this study.

A prevalence of > 90% at *Pfdhps*437**G** was reported in both western Kenya and in this study [10], but this mutation is rare in several countries, which have reported a prevalence of only 0–5% ([22–25]. These results demonstrate the *Pfdhfr* and *Pfdhps* polymorphisms associated with SP resitance are at high frequency among pregnant women in this study population.

The high frequency (94%) of *PfDHPS* gene double mutation (437**G** and 540**E**) reported in this study concurs with findings from other studies in East Africa that reported 90–99% and 99% in western Kenya [10, 26] and Uganda [8], respectively. An average mutation prevalence of 71.3% was seen at codon 540**E** in the coastal regions of Tanzania which was low when compared to the country prevalence of 92.4% [9].

The *Pfdhfr/Pfdhps* quintuple mutant, in either mixed or pure form, is the most clinically relevant molecular marker for SP resistance. In the East African region, the prevalence of molecular markers of SP resistance has been increasing since the emergence of the first resistance-conferring mutations in the 1950s [9], and data from this study shows a great increase among pregnant women living in an malaria endemic zone in coastal Kenya. Continuous molecular surveillance is recommended to determine prevalence of these drug resistant *P. falciparum strains* to build up evidence of reduced susceptibility to the SP drug that result in reduced effectiveness as a prophylactic treatment for malaria in pregnancy. Findings from this study show that there is high prevalence of *Pfdhfr/Pfdhps* haplotype mutations in *P. falciparum strains* isolated from pregnant women which aligns with findings in other parts of Kenya and other tropical regions.

## Conclusion

Results from this study suggest that coastal Kenya has high prevalence of *Pfdhfr* triple mutation and *Pfdhps* double mutation that could potentially undermine the efficacy of SP drug for prophylactic treatment among pregnant women. Despite growing evidence of high prevalence of genetic mutation within the *Pfdhfr* and *Pfdhps* genes associated with SP drug resistance, it is still the recommended drug for IPTp in Kenya and sub-Saharan Africa regions which carry a disproportionately high burden of malaria [2]. There is, therefore, urgent need for development of safe and more effective malaria drugs for prophylactic treatment of malaria in pregnancy. Continuous monitoring and treatment of malaria infection among pregnant women is necessary to avert malaria-related adverse pregnancy outcomes.

## Data Availability

The dataset analysed for this study is available from the corresponding author on reasonable request.
